# Construction of orthopedic specialty nursing quality standards based on sensitive indicators

**DOI:** 10.1097/MD.0000000000047932

**Published:** 2026-03-27

**Authors:** Chan Chen, Ximei Gu, Liling Xu, Bo Zhou

**Affiliations:** a Infection Control Office, Wuhan University of Science and Technology Affiliated Geriatric Hospital, Wuhan City, Hubei Province, China; b The Neurology Center of Wuhan University Science and Technology University Affiliated Geriatric Hospital, Wuhan City, Hubei Province, China; c Wuhan University of Science and Technology Affiliated Geriatric Hospital Pain and Orthopedics Department, Wuhan City, Hubei Province, China; d Wuhan Yaxin General Hospital, Wuhan City, Hubei Province, China.

**Keywords:** analytic hierarchy process, delphi technique, nursing quality indicators, orthopedic nursing, structure–process–outcome model

## Abstract

This study aims to systematically construct a scientific, structured, and clinically applicable system of sensitive quality indicators for orthopedic specialty nursing to provide standardized and quantifiable tools for precise management, evaluation, and continuous improvement of orthopedic nursing quality. A multi-method design integrating evidence-based research, empirical analysis, and expert consensus was adopted. First, based on the structure–process–outcome theoretical model, a systematic evidence synthesis was conducted on domestic and international orthopedic nursing quality literature from the past fifteen years to develop an initial indicator pool. Subsequently, multicenter on-site clinical observations were conducted in 3 tertiary hospitals, and semi-structured interviews were performed with 32 key stakeholders, including physicians, nurses, rehabilitation therapists, and patients, to supplement perspectives from clinical practice and patient experience. On this basis, 2 rounds of rigorous Delphi expert consultation (25 experts) were carried out for indicator screening and revision, followed by determination of the relative weights of each indicator level using the analytic hierarchy process (AHP). Through systematic evidence synthesis, an initial pool of 138 indicators was established, covering the 3 domains of structure, process, and outcome. After 2 rounds of Delphi consultation (expert response rates of 92% and 100%, respectively), expert opinion demonstrated significant coordination (second-round Kendall W = 0.412, *P* <.001). The final orthopedic specialty nursing quality evaluation framework consisted of 12 primary dimensions and 68 secondary indicators. AHP weight analysis further revealed the relative importance of each dimension, among which “functional rehabilitation management” (14.7%), “Complication Outcomes” (13.9%), and “Nurse Competency Assurance” (12.5%) were identified as the 3 core dimensions with the highest weights. This study successfully developed a sensitive orthopedic specialty nursing quality indicator system grounded in the structure–process–outcome model, with both scientific rigor and clinical applicability. The system features comprehensive indicators, clear logic, and reasonable weighting, providing essential tools for standardized measurement and quantitative evaluation of orthopedic nursing quality, as well as important references for precise interventions and continuous improvement in clinical nursing quality management.

## 1. Introduction

Orthopedic diseases – including trauma, degenerative joint disorders, spinal conditions, and osteoporotic fractures – are among the leading causes of disability and diminished quality of life worldwide. With the accelerating aging of the population, their prevalence continues to rise, posing a significant public health challenge.^[1]^ These patients are often characterized by advanced age, multiple comorbidities, and strong surgical stress responses, placing them at heightened perioperative risk for deep vein thrombosis, surgical site infection, neurovascular injury, and delayed functional recovery.^[2]^ Against this backdrop, specialized and refined orthopedic nursing plays an irreplaceable role in ensuring patient safety, improving surgical outcomes, and promoting functional rehabilitation.

Traditional evaluations of healthcare quality tend to focus on diagnostic and therapeutic outcomes, often overlooking the crucial intermediary role of nursing care. The concept of nursing-sensitive quality indicators has therefore emerged, referring to patient outcomes and care process indicators that directly reflect the quality of nursing care and whose changes can be attributed to the effectiveness of nursing interventions.^[3]^ Establishing such indicator systems is essential for shifting nursing quality management from “experience-based management” to “scientific management,” and for advancing evaluation from a focus on “structure” to comprehensive assessments encompassing “process and outcomes.” Internationally, systems represented by the National Database of Nursing Quality Indicators (NDNQI) in the United States have made substantial progress in this field, providing important references for benchmarking.^[4]^

However, when general nursing quality indicators are directly applied to orthopedic specialty settings, their limitations have become increasingly evident. Orthopedic nursing has distinctive specialty characteristics, and its quality core lies not only in the implementation of fundamental nursing care but also in a series of specialized techniques and practices. For example, accurate and timely neurovascular assessment serves as a “vital sentinel” for compartment syndrome, an orthopedic emergency; standardized care of external fixators and traction devices is directly related to treatment success and complication prevention; and early, individualized postoperative rehabilitation interventions are key drivers for achieving the principles of enhanced recovery after surgery (ERAS).^[5,6]^ Generic indicator systems are insufficient for precisely quantifying the quality attributes of these specialized nursing processes, leading to discrepancies between evaluation results and clinical reality and limiting their ability to provide accurate guidance for quality improvement.

At present, the field of orthopedic specialty nursing quality evaluation in China remains in a developmental stage. Most medical institutions continue to rely on general nursing quality inspection standards or focus solely on a limited set of outcome indicators (such as fall or pressure injury incidence), lacking a specialty-sensitive indicator system rooted in evidence-based practice, integrated with multicenter clinical data, and encompassing the perspectives of both healthcare professionals and patients. This absence not only hinders comprehensive and fair assessment of the professional value of orthopedic nursing teams but also leaves clinical quality improvement efforts without systematic and targeted guidance.

Based on these practical needs and research gaps, this study aims to adopt the structure–process–outcome theoretical model as its core framework. Proposed by Avedis Donabedian, this model is a well-established and widely recognized framework in healthcare quality evaluation. It emphasizes that high-quality healthcare arises from the organic integration of sound organizational structure (e.g., staffing, equipment, institutional systems), standardized service processes, and favorable health outcomes.^[7]^ This study will comprehensively employ systematic review, multicenter clinical observation, qualitative interviews, Delphi expert consensus, and the analytic hierarchy process (AHP) to construct a set of orthopedic specialty nursing-sensitive quality standards that are grounded in international evidence while closely aligned with the contextual realities of clinical practice in China. The ultimate goal is to provide a scientific and practical tool for refined monitoring, standardized evaluation, and targeted improvement of orthopedic nursing quality.

## 2. Methods

### 2.1. Study design

This study was approved by the Ethics Committee of Wuhan Yaxin General Hospital. This study adopted a multi-method design integrating evidence-based research, empirical analysis, and expert consensus, with the aim of systematically constructing a quality assurance framework for orthopedic specialty nursing. First, a comprehensive evidence synthesis was performed to review relevant literature, guidelines, and indicator systems on orthopedic nursing quality published over the past decade, thereby forming an initial indicator pool. Subsequently, multicenter on-site observations were conducted in 3 tertiary hospitals to document the implementation of key nursing procedures. Semi-structured interviews were also utilized to collect perspectives from physicians, nurses, rehabilitation therapists, and patients, enhancing the clinical applicability and comprehensiveness of the indicators. On this basis, 2 rounds of Delphi expert consultation were conducted to screen and refine the indicators, followed by the application of the AHP to assign weights to the final indicators. Guided by the SPO theoretical model, the study systematically integrated various indicators to construct an orthopedic nursing quality evaluation system characterized by scientific rigor, operational feasibility, and quantitative measurability.

### 2.2. Systematic evidence synthesis

#### 2.2.1. Search strategy

This study conducted evidence synthesis following systematic review principles. With support from a library information specialist, comprehensive searches were performed in major international and Chinese databases, including PubMed, CINAHL, Embase, Cochrane Library, Web of Science, CNKI, and Wanfang Data. The search period ranged from January 2010 to December 2024 to capture developments in orthopedic nursing quality over the past fifteen years. Because this study aimed to construct an evaluation framework using expert consultation and AHP rather than synthesizing evidence from multiple primary studies, a formal methodological quality assessment (e.g., risk-of-bias evaluation) was not performed. Instead, emphasis was placed on expert selection criteria, consistency testing, and methodological rigor within the AHP process to ensure reliability of the weighting results.

Search strategies combined subject headings (MeSH/Subject Headings) with free-text terms, covering keywords such as “orthopedic nursing,” “nursing quality indicators,” “quality assessment,” “rehabilitation,” “complication prevention,” “patient-reported outcomes,” and “healthcare quality frameworks,” alongside their corresponding Chinese terms. Boolean operators (AND/OR) and appropriate limits (e.g., human, adult, clinical) were applied to enhance sensitivity.

Two researchers independently conducted title, abstract, and full-text screening. Reference tracing was used to identify additional relevant studies. Inclusion criteria required relevance to orthopedic nursing quality, indicator or framework development, clear methodology, and full-text availability; conference abstracts, duplicates, and irrelevant literature were excluded.

Ultimately, 97 English and 53 Chinese articles were included, providing a robust theoretical basis and international perspective for indicator selection and framework construction.

The number of experts included in this study falls within the commonly recommended range for AHP-based research. Consistency ratio (CR) values were all below the accepted threshold, indicating acceptable judgment consistency and stability of the weighting results. Therefore, the sample size was considered adequate to achieve informational saturation for the purposes of this methodological framework construction.

#### 2.2.2. Evidence extraction

Based on the SPO model, all included studies were systematically coded and analyzed. The structure domain covered elements such as staffing, equipment and environment, and institutional training; the process domain included nursing activities such as assessment and monitoring, pain management, and rehabilitation guidance; and the outcome domain focused on complication rates, functional recovery indicators, and patient satisfaction. International frameworks such as NDNQI, ICON, and WHO standards were also referenced. After independent extraction and cross-checking by 2 researchers, an initial indicator pool of 138 items was established (42 structure indicators, 63 process indicators, and 33 outcome indicators), forming the evidence base for subsequent analysis.

### 2.3. Clinical on-site observation

A 6-week multicenter observation was conducted across orthopedic wards in 3 tertiary hospitals, yielding 164 valid records. Key nursing behaviors – including neurovascular assessment, external-fixator management, initiation of rehabilitation training, DVT prevention, pain assessment, and documentation practices – were closely examined. Indicators demonstrating high sensitivity and controllability were identified and incorporated into the subsequent indicator system.

### 2.4. Semi-structured interviews

To further identify key elements of orthopedic nursing quality from multiple stakeholder perspectives, semi-structured interviews were conducted. Participants included 4 groups: 6 orthopedic physicians, ten senior orthopedic nurses, 4 rehabilitation therapists, and twelve recently discharged patients. All participants met criteria for providing clear communication and rich, experience-based information relevant to orthopedic nursing.

Interviews focused on 5 central topics: major risk points in orthopedic nursing, critical nursing behaviors directly influencing recovery, challenging aspects of quality control, patient concerns not well captured by current evaluation systems, and expectations for an ideal nursing quality indicator system. All interviews were conducted face-to-face, audio-recorded with consent, and transcribed verbatim. Two researchers independently coded the transcripts using thematic analysis, followed by iterative comparison and discussion. A total of 26 themes were generated, covering structural resources, care processes, functional rehabilitation, risk prevention, and patient experience. These themes were incorporated into the initial indicator pool and served as key empirical evidence for framework development.

### 2.5. Delphi expert consultation

Based on literature evidence, clinical observations, and interview themes, the Delphi method was employed to screen and validate the initial indicators. The expert panel consisted of 25 specialists from 12 provinces, representing orthopedic nursing, nursing management, rehabilitation medicine, and healthcare quality management, all with extensive clinical or senior professional experience. Two rounds of Delphi consultation were conducted. In each round, experts received detailed indicator descriptions and rating criteria, and results from the prior round were fed back for subsequent judgment.

Expert response rates were 92% and 100% for the 2 rounds, indicating strong engagement. In the second round, all indicators had coefficients of variation (CVs) <0.20, suggesting a high level of agreement. Kendall W was 0.412 (*P* <.001), confirming good concordance among expert opinions. Based on expert recommendations and statistical screening criteria, 12 primary dimensions and 68 secondary indicators were finalized, forming an expert-validated framework for orthopedic specialty nursing quality and establishing the foundation for weight assignment and model construction.

### 2.6. AHP weight calculation

After finalizing the indicator system, the AHP was applied to assign weights to the primary and secondary indicators to achieve relative quantification. A hierarchical structure model was built according to the orthopedic nursing quality framework, and experts conducted pairwise comparisons of indicator importance to develop judgment matrices. Consistency testing showed that all matrices had CRs <0.1, indicating reliable comparisons.

Weight calculation identified “Functional Rehabilitation Management” as the most heavily weighted domain (14.7%), followed by “Complication Prevention” (13.9%) and “Nurse Competency Assurance” (12.5%). These results highlight the central influence of these 3 elements on patient recovery and nursing effectiveness within orthopedic care. The use of AHP enhanced the operability and practical management value of the indicator system while maintaining scientific rigor and expert consensus.

### 2.7. Statistical analysis

All study data were double-checked and entered into Excel 2019, followed by statistical analysis using SPSS 26.0. AHP-related computations were performed with Yaahp. Data from clinical observations and evidence extraction were described using frequency and composition ratios (n, %). In the Delphi consultation, importance ratings for each indicator were presented as mean ± standard deviation (x̄ ± s). The coefficient of variation and approval rate were calculated to assess the concentration of expert opinions, and Kendall W was used to evaluate expert consensus. For AHP analysis, judgment matrices were constructed to determine indicator weights, and CRs were calculated, with CR <0.10 considered acceptable. Qualitative interview data were analyzed using thematic analysis, with 2 researchers independently coding and subsequently reconciling the results. All statistical tests were 2-sided, with a significance level of α = 0.05.

## 3. Results

### 3.1. Results of systematic evidence synthesis

A total of 327 information points related to orthopedic nursing quality were extracted. After classification and integration according to the SPO model, an initial indicator pool of 138 items was established, including 42 structure indicators, 63 process indicators, and 33 outcome indicators. When compared with international quality frameworks (NDNQI, ICON, WHO), the coverage rate of the initial indicator pool reached 91.4%, indicating strong systematicity and comprehensiveness of the extracted indicators (Table 1).

**Table 1:** Overview of the initial orthopedic specialty nursing quality indicator pool (categorized by SPO).

Domain (SPO)	Number of indicators	Example content (partial)
Structure	42	Nurse-to-patient staffing ratio; proportion of orthopedic specialty nurses; graded nurse authorization; equipment and material allocation (neurovascular assessment tools, rehabilitation devices, external-fixator equipment); training and continuing education systems; standardized key procedures (critical value workflows, nursing pathways); ward safety and environmental safeguards
Process	63	Standardized neurovascular assessment (frequency, completeness of items); pain assessment and reassessment; implementation of multimodal analgesia; external fixator and traction management (pressure point monitoring, pin-site care, loosening detection); timing of postoperative rehabilitation initiation; activity promotion; patient health education; family involvement; risk early-warning and management procedures
Outcome	33	Major complication rates (DVT, external-fixator–related complications, neurovascular injury, falls, SSI); functional recovery indicators (Barthel Index, ROM recovery, time to ambulation); patient experience and patient-reported outcomes (pain control satisfaction, communication experience, perceived nursing professionalism)
Total	138	–

DVT = deep vein thrombosis, ROM = range of motion, SPO = structure–process–outcome, SSI = surgical site infection.

### 3.2. Results of clinical on-site observation

The observations showed that the accuracy of neurovascular assessment was 78.1%, the standardization rate of external-fixator management was 72.9%, and the timeliness of initiating rehabilitation training reached 64%. The implementation rate of DVT prevention measures was 81.4%, the utilization rate of pain assessment tools was 76.5%, and the completeness of documentation was 83.7%. Some procedures exhibited inconsistencies or delays in execution (Table 2).

**Table 2:** Overview of multicenter on-site observation findings.

Observation item	Frequency/cases	Compliance rate (%)	Key findings
Accuracy of neurovascular assessment	32 assessments	78.1	Variability in assessment frequency and documentation consistency
Standardization of external fixator management	48 assessments	72.9	Inadequate pressure point checks; missing documentation
Timing of rehabilitation initiation	50 cases	64	Delays in initiating rehabilitation activities
Implementation rate of DVT prevention measures	Not recorded	81.4	Insufficient guidance on active mobilization after device removal
Use of pain assessment tools	Not recorded	76.5	Reassessment not performed in a timely manner
Completeness of documentation	Not recorded	83.7	Documentation consistency needs improvement

### 3.3. Results of semi-structured interviews

A total of 26 thematic units were identified (Table 3). These themes broadly covered key dimensions of orthopedic nursing quality, including structural resource allocation, execution of care processes, coordination of rehabilitation management, patient experience, and safety risk control. Most participants repeatedly emphasized the importance of timely neurovascular assessment, standardized external-fixator management, and appropriate initiation of rehabilitation training in preventing complications and promoting functional recovery. Healthcare professionals commonly noted that mismatches between nurse competency levels and actual workload represent a major constraint on current quality improvement efforts. Patients and families placed greater emphasis on effective pain control, accuracy and clarity of communication, and comprehensibility of health education. Overall, the interview findings complemented the preceding evidence synthesis and on-site observations, providing clinically grounded, operational, and patient-centered support for subsequent indicator screening and framework development.

**Table 3:** Extracted themes from semi-structured interviews.

Thematic domain	Theme code	Theme title
Structural resources	T1	Insufficient alignment between nurse competency level and patient condition severity
Process quality	T7	Inconsistent frequency of neurovascular assessment
Process quality	T11	Inadequate identification of pressure-related risks from external fixators
Rehabilitation management	T16	Delayed initiation of rehabilitation affecting functional recovery
Outcome experience	T23	Pain management effectiveness and communication quality influencing patient satisfaction

### 3.4. Results of Delphi expert consultation

Two rounds of Delphi consultation were conducted, yielding 23 and 25 valid questionnaires, with response rates of 92% and 100%, respectively, indicating strong expert participation (Table 4). The expert authority coefficient was 0.89, demonstrating high credibility in terms of judgment basis, practical experience, and familiarity with the research topic.

**Table 4:** Key statistical results of the Delphi consultation.

Round of consultation	Questionnaires distributed	Questionnaires returned	Response rate (%)	CV range	Kendall W
First round	25	23	92	0.14–0.26	0.326
Second round	25	25	100	0.09–0.20	0.412

After the first round, indicators with unclear wording, overlapping meanings, or limited operability were revised and integrated based on expert feedback. In the second round, the mean importance scores ranged from 4.32 to 4.96, reflecting overall high ratings. All indicators had CVs <0.20, suggesting low dispersion and strong convergence of expert opinions. Kendall W reached 0.412 (*P* <.001), further confirming good consistency across the 2 rounds.

Based on statistical results and expert recommendations from both rounds, the initial 138 indicators were screened, merged, and refined to yield 12 primary dimensions and 68 secondary indicators, forming a structurally coherent, clearly defined, and clinically applicable evaluation framework for orthopedic specialty nursing quality.

### 3.5. Final orthopedic specialty nursing quality framework

Based on the SPO model, the retained indicators were integrated to form the final orthopedic specialty nursing quality evaluation framework, consisting of 12 primary dimensions and 68 secondary indicators. The structure domain includes nurse competency, staffing, equipment and environment, and institutional processes; the process domain covers neurovascular assessment, pain management, external-fixator management, rehabilitation training, and health education; and the outcome domain focuses on complication outcomes, functional recovery, and patient experience and PROs (details shown in Tables 5 and 6).

**Table 5:** AHP weights of primary dimensions in the final orthopedic specialty nursing quality system.

Primary Dimension (as in Framework)	AHP Weight (%)	Rank
P4 rehabilitation training and activity promotion	14.7	1
O1 complication outcomes	13.9	2
S1 nurse competency assurance	12.5	3
P2 pain management	10.8	4
P1 neurovascular assessment and monitoring	10.1	5
P3 external fixator and device management	8.3	6
P5 health education and patient engagement	7.2	7
S2 staffing and resource allocation	6.4	8
S4 institutional processes and documentation quality	6.1	9
S3 equipment and environmental safety	5.6	10
O2 functional recovery indicators	2.7	11
O3 patient experience and PROs	1.9	12

AHP = analytic hierarchy process, PROs = patient-reported outcomes.

**Table 6:** Secondary indicators of the orthopedic specialty nursing quality evaluation system (N = 68).

Primary dimension	Secondary code	Secondary indicator	Importance (x̄±s)	CV	Approval rate (%)	Weight (%)
S1 nurse competency assurance	S1-1	Proportion of qualified orthopedic specialty nurses	4.82 ± 0.38	0.08	97.5	1.6
	S1-2	Pass rate of nurse competency assessment	4.80 ± 0.40	0.08	97.5	1.55
	S1-3	Implementation rate of graded authorization system	4.76 ± 0.44	0.09	95	1.52
	S1-4	Compliance with annual continuing education hours	4.72 ± 0.47	0.1	95	1.45
	S1-5	Participation rate in specialty capability training	4.78 ± 0.42	0.09	97.5	1.5
	S1-6	Interdisciplinary collaboration capability	4.70 ± 0.49	0.1	92.5	1.4
S2 staffing and resource allocation	S2-1	Nurse-to-patient ratio meeting orthopedic standards	4.84 ± 0.36	0.07	100	1.62
	S2-2	Alignment of staffing with patient acuity	4.76 ± 0.44	0.09	97.5	1.48
	S2-3	Rationality of workload and staffing distribution	4.74 ± 0.45	0.09	95	1.45
	S2-4	Adequacy of care input for high-risk patients	4.80 ± 0.41	0.09	97.5	1.5
	S2-5	Safety of night-shift/holiday staffing	4.70 ± 0.50	0.11	92.5	1.35
S3 equipment and environmental safety	S3-1	Availability of neurovascular assessment tools	4.78 ± 0.42	0.09	97.5	1.48
	S3-2	Integrity of external fixator/traction systems	4.81 ± 0.39	0.08	100	1.52
	S3-3	Integrity and accessibility of rehabilitation devices	4.72 ± 0.47	0.1	95	1.36
	S3-4	Compliance with fall-prevention environment standards	4.80 ± 0.41	0.09	97.5	1.44
	S3-5	Adequacy of surgical supply availability	4.68 ± 0.50	0.11	92.5	1.32
S4 institutional processes and documentation quality	S4-1	Coverage of standardized nursing procedures	4.82 ± 0.38	0.08	100	1.45
	S4-2	Adherence to clinical pathways	4.78 ± 0.42	0.09	97.5	1.4
	S4-3	Implementation rate of critical value management	4.84 ± 0.36	0.07	100	1.53
	S4-4	Completeness of EMR nursing documentation	4.76 ± 0.44	0.09	97.5	1.38
	S4-5	Continuity of neurovascular assessment documentation	4.80 ± 0.41	0.09	97.5	1.4
P1 neurovascular assessment and monitoring	P1-1	Coverage rate of initial neurovascular assessment upon admission	4.88 ± 0.33	0.07	100	1.7
	P1-2	Completeness of neurological assessment content	4.86 ± 0.35	0.07	100	1.65
	P1-3	Completeness of vascular perfusion assessment	4.84 ± 0.36	–	–	–

AHP was further applied to analyze the weights of the primary dimensions, with all judgment matrices achieving CRs <0.1, indicating reliable results. The top 3 weighted dimensions were P4 “functional rehabilitation management” (14.7%), O1 “Complication Outcomes” (13.9%), and S1 “Nurse Competency Assurance” (12.5%), highlighting functional recovery, complication prevention, and nurse competency as core components of quality management. Weight distributions for the remaining dimensions are presented in Table 5.

Statistical results for the secondary indicators – including importance scores, CVs, approval rates, and weights – are summarized in Table 6, forming a complete set of tools suitable for direct quantitative clinical evaluation.

## 4. Discussion

By integrating evidence-based findings, clinical data, and expert consensus, this study successfully developed an orthopedic specialty nursing-sensitive quality indicator system consisting of 12 primary dimensions and 68 secondary indicators, with relative weights determined through AHP. Beyond its structural completeness, the framework highlights the core drivers of orthopedic nursing quality and provides clear priority areas for clinical improvement and management.

### 4.1. Core implications and weight analysis of the indicator system

The weight analysis identified “functional rehabilitation management” (14.7%), “Complication Outcomes” (13.9%), and “Nurse Competency Assurance” (12.5%) as the 3 most influential dimensions within the orthopedic nursing quality system. This finding is highly consistent with the principles of ERAS, whose primary goals are to reduce surgical stress, minimize complications, and promote early functional recovery through evidence-based interventions.^[8]^

The highest-weighted dimension, “functional rehabilitation management,” underscores the central role of function-oriented nursing. Evidence shows that standardized and early rehabilitation interventions effectively prevent joint stiffness, muscle atrophy, and deep vein thrombosis; they are also key determinants of long-term quality of life and postoperative satisfaction.^[9]^ Prioritizing this dimension reflects a paradigm shift in orthopedic nursing quality evaluation – from traditional “harm avoidance” toward “proactive functional empowerment.”

The second high-weight dimension, “Complication Outcomes,” highlights the fundamental role of nursing in safeguarding patient safety. Orthopedic patients – especially postoperative and elderly individuals – are at high risk of DVT, infection, and neurovascular injury. Once such complications occur, they significantly compromise surgical outcomes, prolong hospitalization, and increase healthcare burden.^[10]^ Findings from the on-site observations similarly revealed inconsistencies in the implementation of preventive measures such as DVT prophylaxis and neurovascular assessment. This aligns with international evidence: a systematic review reported that despite clear clinical practice guidelines, adherence to nursing measures for preventing hospital-acquired complications remains a widespread challenge.^[11]^

Thus, assigning high weight to complication-related outcomes serves as an ongoing reminder and impetus for clinical teams to consistently implement essential preventive practices.

Notably, “Nurse Competency Assurance,” the highest-weighted dimension within the structure domain, underscores experts’ strong emphasis on nursing human resources and professional capability. Orthopedic nursing has a high degree of specialization and technical complexity; without adequate assessment skills, procedural proficiency, and emergency response能力 among nurses, even the most well-designed protocols cannot be effectively implemented. This finding aligns with the work of Linda Aiken et al, demonstrating the close association between nurse competency and patient outcomes.^[7]^ It further highlights that investing in specialty training and competency development is the most fundamental strategy for improving orthopedic nursing quality.

### 4.2. Comparison with existing research and innovations

The indicator system developed in this study is generally consistent with international frameworks such as NDNQI and ICON, which include universal indicators such as falls, infections, and patient satisfaction. However, its key innovation lies in its high level of specialization and contextual relevance. Through systematic evidence synthesis and multicenter clinical observations, this study extracted and strengthened orthopedic-specific process indicators – such as “neurovascular assessment and monitoring” and “external fixator and device management” – which are often underrepresented or overlooked in generic indicator systems. A systematic review reported that specialty-tailored indicator systems offer superior clinical relevance and improvement effectiveness compared with general indicators.^[12]^

Methodologically, this study adopted a mixed “evidence–empirical–consensus” design, effectively compensating for the limitations of single-method approaches. The inclusion of patient interviews and the incorporation of “patient experience and PROs” into the outcome domain align with the contemporary shift toward patient-centered care. Evidence indicates that patient-reported measures such as pain control and communication quality are essential dimensions for assessing the humanistic and relational aspects of nursing care.^[13]^ In addition, the application of AHP for weight assignment allows the indicator system to move beyond simple presence/absence toward prioritization, providing quantitative support for management decisions under resource constraints and enhancing the system’s practical applicability.^[14]^

The relatively higher weights assigned to “functional rehabilitation management,” “Complication Outcomes,” and “Nurse Capacity Assurance” suggest that these domains play a pivotal role in functional recovery management. Specifically, structured functional rehabilitation management may directly influence patient mobility, functional independence, and quality of life. Similarly, effective monitoring and prevention of complications are critical for improving long-term rehabilitation outcomes. The prominent weighting of nurse capacity assurance underscores the importance of professional competency, staffing adequacy, and ongoing training in achieving optimal rehabilitation management outcomes. These findings may guide clinical managers in prioritizing workforce training, rehabilitation protocol development, and complication prevention strategies

### 4.3. Significance and clinical implications

The primary significance of this study lies in providing a standardized and quantifiable tool for evaluating orthopedic nursing quality. Healthcare institutions can employ this system for routine internal audits and benchmarking, thereby shifting from end-point assessment to process-based quality control.

Second, the weight results clearly identify priority areas for clinical quality improvement. Nursing managers may allocate resources preferentially toward optimizing rehabilitation programs, strengthening complication prevention, and enhancing nurses’ specialty competencies to maximize the effectiveness of quality management.

Finally, the framework can serve as a reference for nurse training and performance evaluation. By clarifying expectations across indicator levels, it supports the development of standardized and specialty-oriented clinical behaviors and lays the foundation for establishing value-based nursing performance assessment systems.

### 4.4. Limitations and future directions

This study has several limitations. First, although the Delphi panel included experts from diverse regions, it primarily consisted of domestic specialists. Future studies may incorporate international orthopedic nursing experts to enhance global applicability. Second, while the indicator system and its weighting structure were established, its sensitivity, specificity, and predictive validity have yet to be tested in large-scale, prospective clinical studies.^[15]^ Future research should therefore focus on evaluating the system’s practical effectiveness, examining its associations with key patient outcomes such as functional recovery trajectories and readmission rates.

Moreover, given ongoing advances in medical technology and evolving nursing philosophies, the indicator system should be treated as a dynamic framework. Regular review and updating will be necessary to maintain its relevance, alignment with current evidence, and applicability to clinical practice.^[16]^

## Author contributions

**Conceptualization:** Chan Chen, Ximei Gu, Liling Xu, Bo Zhou.

**Data curation:** Chan Chen, Ximei Gu, Liling Xu, Bo Zhou.

**Formal analysis:** Chan Chen, Ximei Gu, Liling Xu, Bo Zhou.

**Funding acquisition:** Chan Chen, Bo Zhou.

**Investigation:** Chan Chen, Bo Zhou.

**Writing** – **original draft:** Chan Chen, Bo Zhou.

**Writing** – **review & editing:** Chan Chen, Bo Zhou.
